# Electrochemical characterization of Z-scheme charge transfer in biomass-derived ZnO/carbon dots for efficient tetracycline degradation†

**DOI:** 10.1039/d5ra02606g

**Published:** 2025-07-14

**Authors:** Wan Nuraishah Wan Ishak, Huey Ling Tan, Noor Fitrah Abu Bakar, Norbert Radacsi, Ying Pei Lim

**Affiliations:** a Faculty of Chemical Engineering, Universiti Teknologi MARA 40450 Shah Alam Selangor Malaysia hueyling@uitm.edu.my; b School of Engineering, Institute for Materials and Process, The University of Edinburgh King's Buildings Edinburgh EH9 3FB UK; c School of Engineering, Institute for Bioengineering, The University of Edinburgh Mayfield Road Edinburgh EH9 3JL UK; d Centre for Cardiovascular Science, The Queen's Medical Research Institute (QMRI), University of Edinburgh BioQuarter, 47 Little France Crescent Edinburgh EH16 4TJ UK N.Radacsi@ed.ac.uk

## Abstract

This study investigates the electrochemical properties of a zinc oxide/carbon dots (ZnO/CDs) nanocomposite synthesized *via* a hydrothermal route, where biomass-derived CDs enhance charge separation and extend visible-light absorption. X-ray photoelectron spectroscopy reveals strong interfacial interactions between ZnO and CDs, facilitating efficient charge transfer. The ZnO/CDs (1 : 2) nanocomposite demonstrated superior tetracycline degradation efficiency of 86.6% under visible light, significantly outperforming pristine ZnO and other composite ratios. To elucidate the enhanced charge carrier dynamics, a comprehensive electrochemical analysis was conducted. Electrochemical impedance spectroscopy showed a smaller semicircle diameter in the Nyquist plot, indicating a lower interfacial resistance, which suggests improved electron mobility. Cyclic voltammetry and transient photocurrent measurements further confirmed enhanced charge separation, while Mott–Schottky analysis verified a Z-scheme charge transfer mechanism, effectively suppressing electron–hole recombination. Reactive species trapping experiments identified ˙OH and O_2_˙− radicals as the dominant active species responsible for photocatalytic degradation. The photocatalyst exhibited remarkable recyclability and reusability, maintaining 85% of its photocatalytic activity over five cycles, demonstrating its stability and practical applicability for wastewater remediation. This study underscores the pivotal role of biomass-derived CDs in modulating the electronic properties of ZnO, offering a sustainable and efficient strategy for environmental cleanup.

## Abstract 2

This study investigates the electrochemical properties of a zinc oxide/carbon dots (ZnO/CDs) nanocomposite synthesized *via* a hydrothermal route, where biomass-derived CDs enhance charge separation and extend visible-light absorption.

## Graphical abstract

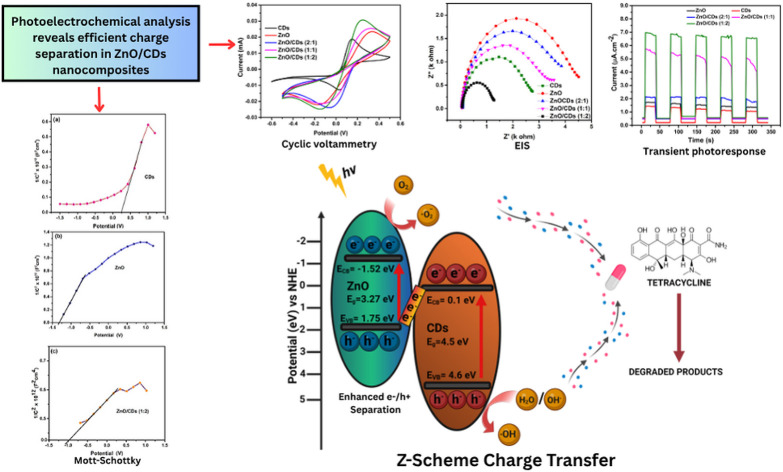

## 1. Introduction

Antibiotic pollution has emerged as a critical environmental threat, largely driven by the widespread use of broad-spectrum antibiotics such as tetracycline (TETR) in both human and veterinary medicine.^1^ Inadequate removal during wastewater treatment leads to the persistent release of TETR and its derivatives into surface and groundwater, as well as marine environments.^2^ Even at trace levels, these contaminants exert significant selective pressure that promotes the proliferation of antibiotic-resistant bacteria (ARB) and the spread of antibiotic resistance genes (ARG).^2^ This phenomenon poses a serious threat to both human and environmental health, contributing to the global crisis of antimicrobial resistance (AMR). Recognizing this critical issue, the World Health Organization (WHO) has recently issued its first guidance on managing antibiotic pollution from manufacturing, underscoring the crucial role of environmental contamination in fueling the AMR crisis.^3^ This guidance emphasizes the urgent need for robust regulatory frameworks and innovative mitigation strategies to address this pressing environmental and public health challenge. Furthermore, antibiotic contamination of water resources has emerged as a critical 21st-century environmental challenge, posing significant risks to both the environment and human health. According to previous study,^4^ the global mortality rate due to antibiotic resistance could surpass that of HIV or malaria if the current trajectory continues. This alarming trend arises from the evolution of antimicrobial resistance in bacteria, fungi, viruses, and parasites, rendering them resistant to drugs. This resistance not only complicates disease treatment but also increases the likelihood of disease transmission, leading to increased fatalities and mortality. Despite its widespread use, TETR exhibits significant recalcitrance to conventional wastewater treatment processes due to its stable chemical structure and resistance to biological degradation. This necessitates the exploration of alternative treatment technologies.^5^ Thus, photocatalytic degradation has emerged as a promising approach owing to its high efficiency and potential for sustained operation compared to conventional methods. While traditional photocatalysts such as TiO_2_ and ZnO can degrade TETR under UV irradiation, their limited visible light absorption and rapid electron–hole recombination, severely curtail their practical applications.^6^

Zinc oxide (ZnO), in particular, has attracted considerable attention as a photocatalyst due to its high stability, low cost, and favorable photochemical properties.^7^ Nonetheless, its intrinsic wide bandgap (3.27 eV) restricts light absorption to the ultraviolet region, while rapid charge carrier recombination further impedes its photocatalytic efficiency.^7^ To overcome these limitations, this study explores a novel strategy by integrating carbon dots (CDs) with ZnO, forming a heterostructure that enhances visible light absorption and improves photocatalytic degradation of TETR. CDs exhibit unique optical and electronic properties, including strong photoluminescence, excellent electron transfer capabilities, and up-conversion luminescence. These properties enable CDs to absorb light in the visible spectrum and efficiently transfer energy to ZnO, thereby extending its absorption range from the ultraviolet to the visible region.^8^

Building on our prior findings, this study continues to explore the potential of CDs derived from biomass waste, specifically oil palm empty fruit bunches (EFB), as a sustainable and effective means to enhance the photocatalytic performance of ZnO. While previous studies,^7,9,10^ have demonstrated the structural, optical, and morphological properties of the ZnO/CDs composite using PL, UV-DRS, XRD, FTIR, and SEM-EDX analyses, the critical role of CDs in suppressing electron–hole recombination and enhancing charge separation within ZnO/CDs heterostructures remains underexplored. This study aims to bridge this knowledge gap by investigating the charge recombination dynamics in the ZnO/CDs nanocomposite. To achieve this, electrochemical analyses such as electrochemical impedance spectroscopy (EIS), Mott–Schottky analysis, and transient photocurrent response are employed to provide a comprehensive understanding of the charge transfer resistance, energy band alignment, and charge separation efficiency of the ZnO/CDs composite. These advanced electrochemical techniques are crucial for elucidating the charge carrier dynamics that were not fully addressed in previous studies.^7,9,10^ The insights from the electrochemical study provide a more detailed mechanistic understanding of the ZnO/CDs Z-scheme heterostructure.

Moreover, Z-scheme heterojunctions reported in the previous studies exhibit higher charge separation efficiency, resulting in improved photocatalytic activity compared to photocatalytic mechanisms.^11^ For instance, Zhang *et al.* fabricated a Z-scheme heterojunction of SnS_2_/In_2_S_3_*via* a one-pot hydrothermal synthesis approach, which demonstrated efficient charge carrier separation. The heterostructure exhibited a remarkable 99.2-fold and 15.3-fold enhancement in Rhodamine B (RhB) photodegradation activity compared to pristine SnS_2_ and In_2_S_3_, respectively.^12^ Meanwhile, Liu *et al.* synthesized a BiVO_4_/Bi_25_VO_40_ heterojunction through an alkali-mediated dissolution–recrystallization strategy, which enabled atomic-scale interfacial coupling to establish a Z-scheme band alignment.^11,13^ The intimate heterostructure promoted efficient charge migration and spatial separation of electron–hole pairs *via* suppressed recombination kinetics, leading to a marked improvement in photocatalytic tetracycline degradation (8.2-fold enhancement over pristine BiVO_4_).^14^

In this study, a novel ZnO/CDs nanocomposites Z-scheme heterostructure was synthesized and comprehensively characterized. Techniques such as EIS, Mott–Schottky analysis, and transient photocurrent response were employed to elucidate the photoelectrochemical properties of the composite. Initial photocatalytic activity of the ZnO/CDs nanocomposite was evaluated under UV light irradiation to establish a baseline for comparison. Subsequently, photocatalytic performance was further investigated under visible and sunlight illumination. Trapping tests were conducted to identify the dominant reactive species involved in the degradation process. Furthermore, LC-MS analysis was utilized to elucidate the degradation pathway of tetracycline. These investigations offer a cost-effective and scalable solution for mitigating antibiotic pollution, thereby contributing to sustainable wastewater treatment and the global effort to curb antimicrobial resistance.

## 2. Materials and methods

### 2.1. Materials and chemicals

Carbon dots (CDs) were synthesized from Empty Fruit Bunches (EFB), a lignocellulosic biomass sourced from a Malaysian oil palm plantation in Kedah. Chemicals, including zinc(ii) acetate dihydrate Zn (CH_3_COO)_2_·2H_2_O, >98%, ethanol (CH_3_CH_2_OH, >95%), and sodium hydroxide (NaOH, >98%), were supplied by Sigma-Aldrich. Tetracycline (TETR > 98%) antibiotics were supplied by Merck Millipore. All reagents were of analytical grade and used as received without further purification. UV irradiation was provided by Kintons KT6-UVC lamps (2 × 15 W), while visible light illumination was supplied by an 80 W lamp. Both light sources were purchased from SA Fish World Sdn Bhd, Malaysia.

### 2.2. Synthesis of zinc oxide

**Fig. 1:**
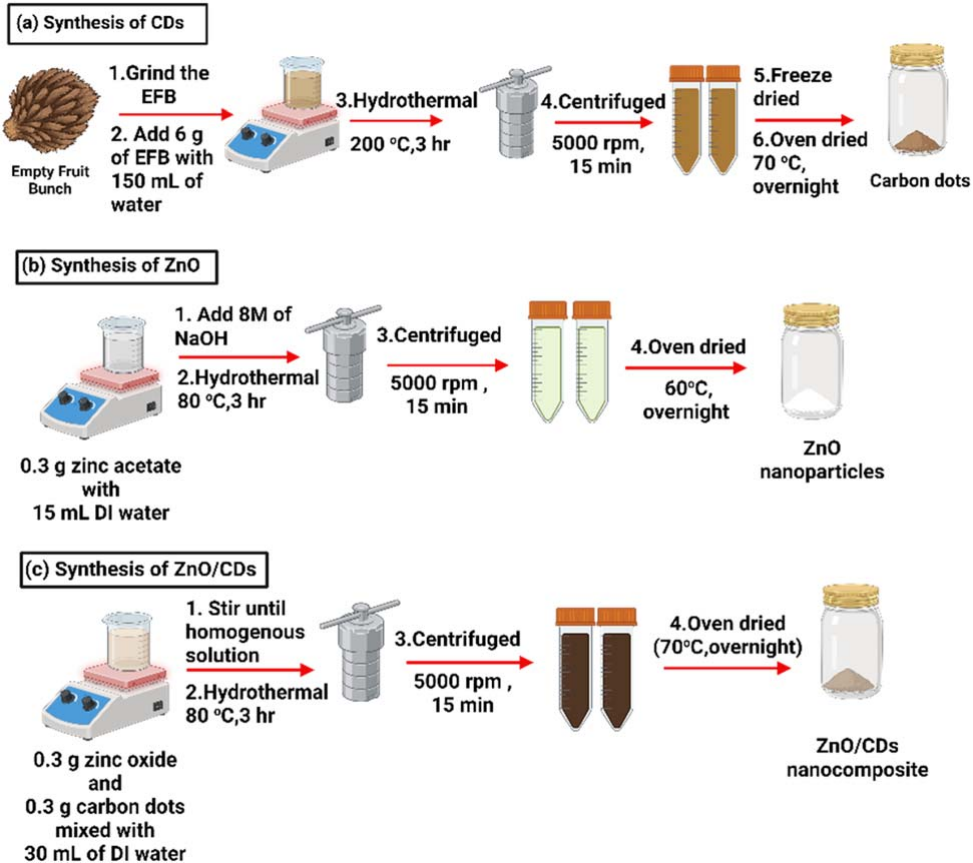
Schematic illustration of the hydrothermal synthesis of ZnO/CDs nanocomposite: (a) CDs from EFB, (b) ZnO nanoparticles, (c) ZnO/CDs nanocomposites (2 : 1, 1 : 1, 1 : 2).

ZnO nanoparticles were synthesized *via* a one-step hydrothermal method, following the procedures reported in previous studies,^9,15^ as depicted in Fig. 1b. Briefly, 0.3 g of zinc acetate was dissolved in 15 mL of deionized water, and the pH of the solution was adjusted to 11 by the gradual addition of 8 M NaOH under vigorous stirring. The resulting mixture was transferred into a 150 mL Teflon-lined autoclave and subjected to hydrothermal treatment at 80 °C for 3 hours. Upon completion, the precipitate was collected by centrifugation at 5000 rpm for 15 minutes and washed with ethanol and deionized water (3 : 1) to eliminate impurities, dried at 60 °C, and ground into a fine powder for further use.

### 2.3. Synthesis of zinc oxide/carbon dots

Carbon dots were synthesized *via* a hydrothermal method.^9,16^ Briefly, 6 g of EFB powder was dispersed in 150 mL of deionized water and subjected to hydrothermal treatment at 200 °C for 3 hours within a Teflon-lined autoclave. Subsequent purification involved centrifugation at 5000 rpm for 15 minutes, followed by filtration through a 0.22 μm nylon syringe filter. The resulting solution was then freeze-dried and further dried in an oven at 70 °C overnight (Fig. 1a). For the preparation of ZnO/CDs nanocomposites, 0.3 g of ZnO nanoparticles were mixed with 0.3 g of CDs in a 1 : 1 of ratio and dispersed in 30 mL of deionized water, followed by stirring for 1 hour. Then, the homogeneous mixture was subjected to hydrothermal treatment at 80 °C for 3 hours in a Teflon-lined autoclave. The resulting precipitation was washed four times with ethanol and deionized water with ratio 3 : 2 (v/v), dried at 70 °C, and ground into a fine powder. This process was repeated to obtain ZnO/CDs nanocomposites with ratios of 1 : 2 and 2 : 1 (Fig. 1c).

### 2.4. Characterization of photocatalyst

The surface composition and chemical states were conducted by X-ray photoelectron spectroscopy (XPS, Kratos Axis Ultra-DLD system) equipped with Al Kα (1486.6 eV). Electrochemical measurements were conducted using an Autolab PGSTAT302N potentiostat in a three-electrode configuration, employing carbon and gold screen-printed electrodes. Two types of screen-printed electrodes (SPEs) from Metrohm DropSens were employed depending on the sample. The DRP-110 SPE (carbon working electrode, carbon counter electrode, and Ag/AgCl reference electrode) was used for ZnO and ZnO/CDs composites, while the DRP-220AT SPE (gold working electrode, carbon counter electrode, and Ag/AgCl reference electrode) was specifically used for carbon dots. Prior to measurement, the working electrode surface was drop-cast with the photocatalyst material and dried at room temperature. Mott–Schottky plots and transient photocurrent density measurements were performed in a 0.1 M Na_2_SO_4_ electrolyte to evaluate the semiconductor properties and photo response. Cyclic voltammetry (CV) and electrochemical impedance spectroscopy (EIS) were conducted using a 0.1 M KCl solution containing 5 mM K_3_[Fe(CN)_6_]/K_4_[Fe(CN)_6_] (1 : 1) as the redox probe electrolyte to investigate the electrochemical characteristics of the synthesized materials.

### 2.5. Photodegradation of tetracycline

Photocatalytic degradation of tetracycline (TETR) was initially performed under UV irradiation (365 nm) from a lamp positioned 15 cm above a suspension containing 0.05 g ZnO/CDs photocatalyst in 50 mL of a 15 ppm TETR solution. Prior to UV exposure, the suspension was stirred in the dark for 30 minutes to achieve adsorption–desorption equilibrium. Following irradiation for predetermined durations, the mixture was filtered (0.22 μm syringe filter), and the residual TETR concentration was quantified using UV-Vis spectrophotometry at 357 nm based on a calibration curve. To compare UV-induced degradation, subsequent experiments were conducted under sunlight (40 000–80 000 lux intensity, clear skies, 12:00–2:00 PM) and visible light irradiation from an 80 W lamp emitting at 400 nm. The degradation efficiency was determined using eqn (1),^6^ considering initial (*C*_0_) and final (*C*_*t*_) TETR concentrations.1
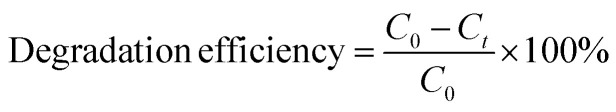


### 2.6. Trapping experiment

The identification of the dominant reactive oxygen species (ROS) involved in TETR degradation was achieved by subjecting the reaction mixture to visible irradiation in a presence of specific scavengers over a period of 180 minutes. To identify the respective roles of these ROS, 1 mM of various scavengers were spiked into the reaction system.^17–19^*tert*-Butanol (BuOH) selectively quenched hydroxyl radicals (˙OH), while ethylenediaminetetraacetic acid disodium salt (EDTA) scavenged positively charged holes (h^+^). Silver nitrate (AgNO_3_) functioned as an electron scavenger (e^−^), and finally, *p*-benzoquinone (*p*-BQ) was used to trap superoxide anion radicals (˙O_2_^−^).

### 2.7. Photostability and re-usability of ZnO/CDs nanocomposites

The photostability and reusability of the ZnO/CDs nanocomposite photocatalyst were systematically assessed across five consecutive cycles of TETR degradation. After each cycle, the photocatalyst was recovered by centrifugation using a 3 : 2 (v/v) ethanol–water mixture, followed by thorough washing to remove residual contaminants. Subsequently, the recovered photocatalyst was dried overnight at 60 °C to restore its activity for the subsequent degradation cycle. This process was repeated for five cycles to assess the consistency of its photocatalytic performance.^9^ This process was repeated for five cycles to assess the consistency of its photocatalytic performance. High-resolution transmission electron microscopy (HRTEM) and XRD (X-ray diffraction) were used to investigate any potential alterations induced by repeated use.

## 3. Results and discussion

### 3.1. Structural and morphological characteristics

CDs were synthesized from oil palm empty fruit bunch (EFB) *via* hydrothermal treatment at 200 °C for 3 hours. This duration was selected based on prior studies indicating that moderate reaction times are sufficient to induce effective carbonization and polymerization of biomass precursors, yielding CDs with favorable photoluminescence and structural stability.^20^ During hydrothermal processing, the EFB undergoes dehydration, aromatization, and carbonization, resulting in the formation of carbonaceous cores and surface functional groups that govern the optical behavior of CDs.^21^ Extended reaction times (typically 6–25 h) can enhance graphitization and narrow the band gap, resulting in red-shifted emission and improved visible-light absorption.^20,22–25^ However, excessively long durations may cause particle aggregation, fluorescence quenching, or over-carbonisation, which can reduce quantum yield and aqueous dispersibility of CDs.^21,26^

The successful synthesis of ZnO/CDs was verified through XRD, and HRTEM analyses (ESI, Fig. S1 and S2†). The XRD patterns confirmed the crystalline structure of ZnO with no detectable impurity phases, while HRTEM images (ESI, Fig. S1†) revealed a well-dispersed distribution of carbon dots on ZnO, which facilitates charge transfer at the interface. Additionally, FTIR analysis (ESI, Fig. S3†) provided further confirmation of the presence of functional groups associated with carbon dots, supporting their successful integration into the ZnO matrix.

### 3.2. XPS analysis: chemical states and charge transfer

**Fig. 2:**
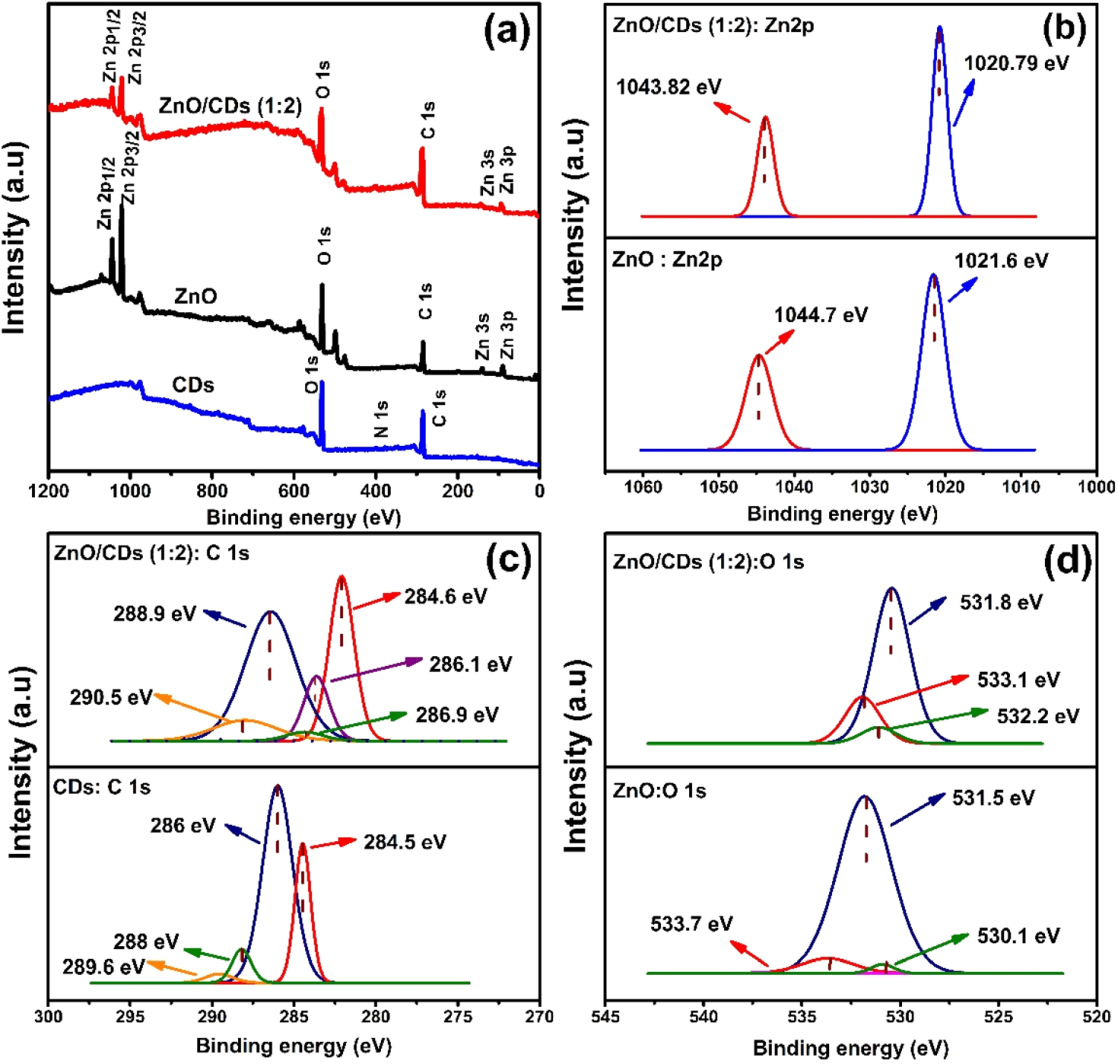
XPS spectra of (a) survey scans, (b) Zn 2p core level spectra, (c) C 1s core level spectra, and (d) O 1s core level spectra for ZnO, CDs, and the ZnO/CDs (1 : 2) composite.

XPS analysis was employed to investigate the surface chemical composition and electronic states of ZnO, CDs, and the ZnO/CDs (1 : 2) nanocomposites. Survey spectra (Fig. 2a) confirmed the presence of expected elements: C, O, and N in CDs; Zn and O in ZnO; and all four elements in the composite, validating the successful incorporation of CDs into the ZnO matrix. The Zn 2p region (Fig. 2b) for ZnO nanoparticles exhibits characteristic spin–orbit split peaks at 1021.6 eV (Zn 2p_3/2_) and 1044.7 eV (Zn 2p_1/2_), consistent with Zn^2+^ in ZnO nanoparticles.^7^ The energy separation of 23.1 eV between these peaks confirms the +2 oxidation state of zinc.^10^ Upon CDs incorporation, these peaks shift to 1020.79 eV (Zn 2p_3/2_) and 1043.82 eV (Zn 2p_1/2_) in the composite, a shift of approximately 0.8 eV toward lower binding energies, suggesting a change in the electronic environment of Zn^2+^ due to the interaction with the CDs, potentially indicating charge transfer or the formation of Zn–O–C bonds at the interface.^27–29^ In the high-resolution of C 1s spectra (Fig. 2c), there are four peaks observed at 284, 286, 288, and 289 eV for CDs, which correspond to C=C/C–C, C–N, C=O, and O–C=O, respectively.^7^ While for ZnO/CDs (1 : 2), a new peak is observed at 286.1 eV, which shows that the incorporation of CDs and ZnO was successful. For the O 1s spectra of ZnO and ZnO/CDs (1 : 2) nanocomposites (Fig. 2d), distinct peaks represent various oxygen species and defects. In pristine ZnO, the peak at 531.5 eV corresponds to surface oxygen vacancies (O_vac_), while the peaks at 533.7 eV and 530.1 eV are attributed to oxygen in hydroxyl groups (O–H) and lattice oxygen, respectively. In the ZnO/CDs (1 : 2) nanocomposite, shifts in binding energies and variations in peak intensities are observed, indicating strong interfacial interactions between ZnO and CDs. The peak corresponding to oxygen vacancies shifts to 531.8 eV in the composite and shows increased intensity, indicating that the addition of CDs promotes the formation of more oxygen vacancies during the synthesis process.^10^ Oxygen vacancies are known to play a crucial role in photocatalysis by trapping photogenerated electrons and facilitating charge separation.^10^ Additionally, the peaks at 533.1 eV and 532.2 eV in the composite are assigned to hydroxyl oxygen and adsorbed oxygen species, respectively. The higher concentration of oxygen vacancies in ZnO/CDs enhances surface activity, facilitates reactant adsorption, and narrows the band gap, thereby improving light absorption and photocatalytic performance.^10^ These results demonstrate the significant role of CDs in optimizing the structural and surface properties of ZnO. The overall changes in the characteristic peaks of C 1s, and O 1s high-resolution spectra further provide evidence supporting the successful preparation of ZnO/CDs.

### 3.3. Optical properties

**Fig. 3:**
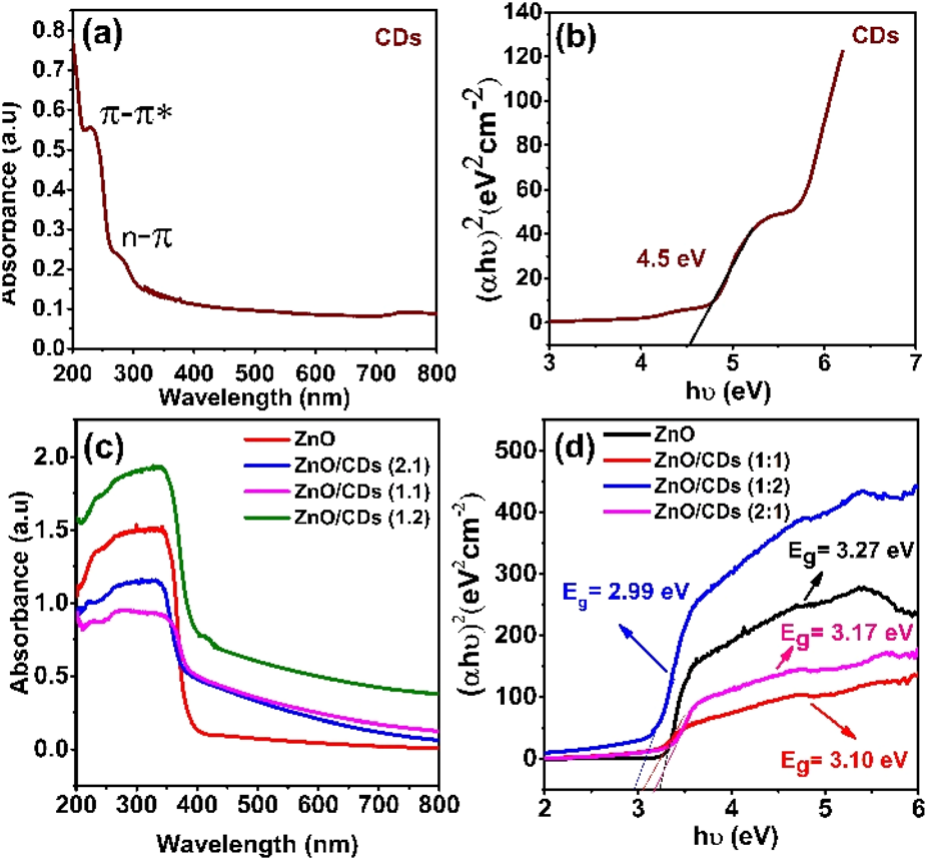
(a and c) UV-DRS and (b and d) plot of (*αhν*)^2^*versus* photon energy of the of CDs, ZnO nanoparticles and ZnO/CDs composites.

The UV-vis absorption spectrum in Fig. 3b showed that CDs possess a wide band gap 4.5 eV, indicating limited π-conjugation in the carbon core, likely due to quantum confinement and surface passivation effects (Fig. 3a and b).^30–32^ Although absorption is mainly in the UV region, surface defect states introduce mid-gap levels that enable visible-light absorption.^33–35^ Upon integration with ZnO, the band gap narrowed to 2.99 eV for ZnO/CDs (1 : 2), as shown in Fig. 3d, indicating strong electronic interactions and enhanced visible-light harvesting. This band structure modulation enhances charge separation, as further supported by time-resolved photoluminescence (TRPL) analysis. ZnO/CDs (1 : 2) nanocomposite exhibited a significantly shorter average carrier lifetime (2.084 ns) compared to pristine ZnO (4.198 ns) (see Table S1 of ESI†), suggesting suppressed radiative recombination.^32^ This shortened lifetime implies that photoexcited electrons from CDs are efficiently transferred to ZnO to participate in redox reactions, thereby enhancing the photocatalytic performance.^31,32,36^

The fluorescence quantum yield, defined as the ratio of emitted photons to absorbed photons, was determined using the well-established comparative method.^37^ This method involves comparing the wavelength-integrated fluorescence intensities of the test sample with a suitable standard exhibiting an overlapping excitation wavelength range. To enhance the accuracy of quantum yield determination, the influence of absorbance on the integrated fluorescence intensity was considered for both the test sample and the standard. The quantum yield (QY) of CDs was determined by comparing the optical properties of CDs to the standard quinine sulfate solution 0.1 M H_2_SO_4_ which has a QY of 54.6% at an excitation wavelength of 350 nm. The QY of the CDs samples was calculated by using the following eqn (2).2
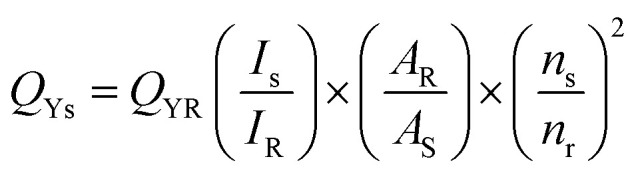
where R and S are the reference (quinine sulphate) and sample (carbon dots), *Q*_y_ is the quantum yield, *I* is PL area under the graph and *n* is refractive index where *n* of sample and reference are 1.294 and 1.33 respectively.^38^ The quantum yield of CDs obtained in this study was compared with the quantum yield of CDs derived from other natural precursors, and their quantum yields are listed in Table 1. The results distinctly demonstrate that the use of EFB in this study significantly improved the quantum yield, achieving a notable enhancement compared to other waste-derived precursors. This highlights the exceptional potential of EFB as a superior and sustainable carbon source for high-performance CDs. As shown in Fig. 3d, pristine ZnO nanoparticles exhibited photocatalytic activity only under UV light due to their wide bandgap (3.27 eV), which restricts light absorption to the ultraviolet region. However, the incorporation of CDs significantly extended the optical absorption of the ZnO/CDs composite into the visible light region. This enhancement is attributed to the high quantum yield of CDs, which enables effective visible light absorption and facilitates energy transfer to ZnO, thereby activating the composite under visible light irradiation. Consequently, the ZnO/CDs heterostructure demonstrated superior photocatalytic degradation of TETR under visible light compared to pristine ZnO, highlighting the pivotal role of CDs in enhancing light utilization and charge separation efficiency.

**Table 1:** Comparison of fluorescence quantum yields of carbon dots synthesized from different precursors

Carbon precursors from waste	Quantum yield (%)	References
Soybean	25	7
Magnolia flower	8.13	39
Onion waste	28	40
Dried lemon peels	22.3	41
Waster carbon paper	8.5	42
Orange waste peels	11.37	43
Lemon juice	14.86	44
Empty fruit bunch	30	This study

### 3.4. Photoelectrochemical properties

**Fig. 4:**
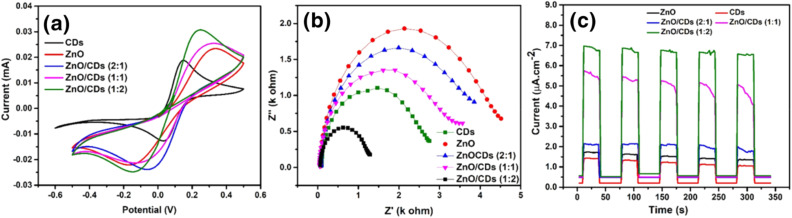
Electrochemical characterization of ZnO, CDs, and ZnO/CDs nanocomposites (2 : 1, 1 : 1, 1 : 2) (a) cyclic voltammetry; (b) Nyquist plots; (c) photocurrent response.

Cyclic voltammetry (CV) and electrochemical impedance spectroscopy (EIS) were employed to investigate the charge transfer behavior of the synthesized catalysts. CV measurements were conducted using a UV lamp as the light source and a scan rate of 100 mV s^−1^. A higher oxidation–reduction reaction (ORR) peak current in the CV spectrum generally indicates improved charge transfer efficiency (Fig. 4a). The CDs exhibited minimal current response, consistent with their role as charge mediators rather than active redox participants. In contrast, ZnO displayed moderate redox activity with broad peaks, indicating limited electron transfer kinetics due to charge carrier recombination.^45^ Conversely, the ZnO/CDs nanocomposites exhibited significantly enhanced current densities and well-defined redox peaks, attributed to the synergistic interaction between ZnO and CDs, where the latter facilitated efficient charge separation and suppressed recombination.^46^ At 0.2 V, the current density of the ZnO/CDs (1 : 2) composite is approximately 2.5 times higher than that of pristine ZnO. This significant increase suggests that the ZnO/CDs 1 : 2 composite exhibits superior electron transfer efficiency compared to the other ratios (Fig. 4a). The enhanced performance is likely due to the optimal distribution of CDs within the ZnO matrix at this ratio, which facilitates efficient electron transport. Meanwhile EIS results from the Nyquist plots (Fig. 4b) show that pristine ZnO exhibits the highest impedance, as indicated by the largest semicircle, consistent with its characteristically poor electrical conductivity.^10^ The incorporation of CDs into the ZnO matrix leads to a significant reduction in charge transfer resistance, indicating improved electrical conductivity and an enhanced charge separation efficiency. Notably, the ZnO/CDs (1 : 2) composite demonstrates the smallest semicircle among all the tested samples in the Nyquist plot, reflecting the lowest resistance and, consequently, the most efficient charge transport properties. This enhancement is attributed to the synergistic effect between ZnO and CDs, where CDs introduce highly conductive pathways within the composite structure, effectively boosting electron mobility and reducing the overall charge transfer resistance. These improvement in conductivity and charge transport are consistent with the CV analysis, further supporting the conclusion that the ZnO/CDs (1 : 2) composite possesses superior photocatalysis performance. To further investigate the photoresponse behavior of ZnO, CDs, and ZnO/CDs composites with different ratios, transient photocurrent response (*I*–*t*) were conducted under periodic light on/off cycles, as shown in Fig. 4c. Pristine ZnO exhibits the lowest photocurrent response due to rapid charge carrier recombination and limited light absorption.^47^ The incorporation of CDs significantly enhances the photocurrent, indicating improved charge separation and transport. Among the composites, ZnO/CDs 1 : 2 demonstrates the highest and most stable photocurrent response, suggesting superior photogenerated charge carrier dynamics. This enhancement is attributed to the role of CDs as electron acceptors, which facilitate efficient charge transfer.^10^ The repetitive and consistent photocurrent cycles further confirm the excellent photostability of the materials.

### 3.5. Energy band structure

**Fig. 5:**
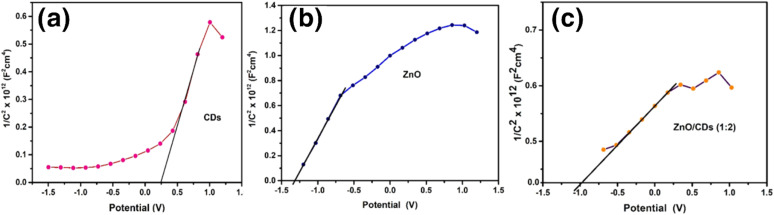
Mott–Schottky plots for (a) CDs, (b) ZnO, and (c) ZnO/CDs composites.

The Mott–Schottky analysis confirmed the n-type semiconducting behavior of all materials, with ZnO exhibiting the highest carrier concentration, followed by the ZnO/CDs composite and CDs as shown in Fig. 5a–c. The flat band potentials (Efb) were determined as 0.3 V (CDs), −1.52 V (ZnO), and −1.2 V (ZnO/CDs) *versus* Ag/AgCl. After conversion to the normal hydrogen electrode (NHE) scale, the corresponding conduction band (CB) edge positions were 0.497 V (CDs), −1.323 V (ZnO), and −1.003 V (ZnO/CDs), while the valence band (VB) positions were 4.997 V, 1.947 V, and 1.987 V (*vs.* NHE), respectively. The significant shift in Efb for ZnO/CDs suggests the formation of a Z-scheme charge transfer mechanism (Fig. 7), wherein electrons from the conduction band (CB) of CDs recombine with holes from the valence band (VB) of ZnO. This process retains the highly reducing electrons in the ZnO CB and the strongly oxidizing holes in CDs VB, effectively enhancing charge separation. As a result, the generation of reactive oxygen species (ROS), such as superoxide radicals (˙O_2_^−^) and hydroxyl radicals (˙OH), is confirmed by scavenger experiments that showed reduced photocatalytic activity upon radical inhibition. Further validation of the Z-scheme mechanism is supported by photoluminescence (PL) quenching (ESI Fig. S4†), extended carrier lifetime from time-resolved photoluminescence (TRPL) measurements (ESI, Table S1†), reduced charge transfer resistance from EIS analysis (Fig. 4b), and enhanced photocurrent response (Fig. 4c). These findings collectively confirm suppressed charge recombination and improved charge mobility, thereby substantiating the efficient Z-scheme mechanism (Fig. 7), and its role in achieving superior photocatalytic performance of the ZnO/CDs nanocomposites for environmental remediation applications.

### 3.6. Trapping experiment

**Fig. 6:**
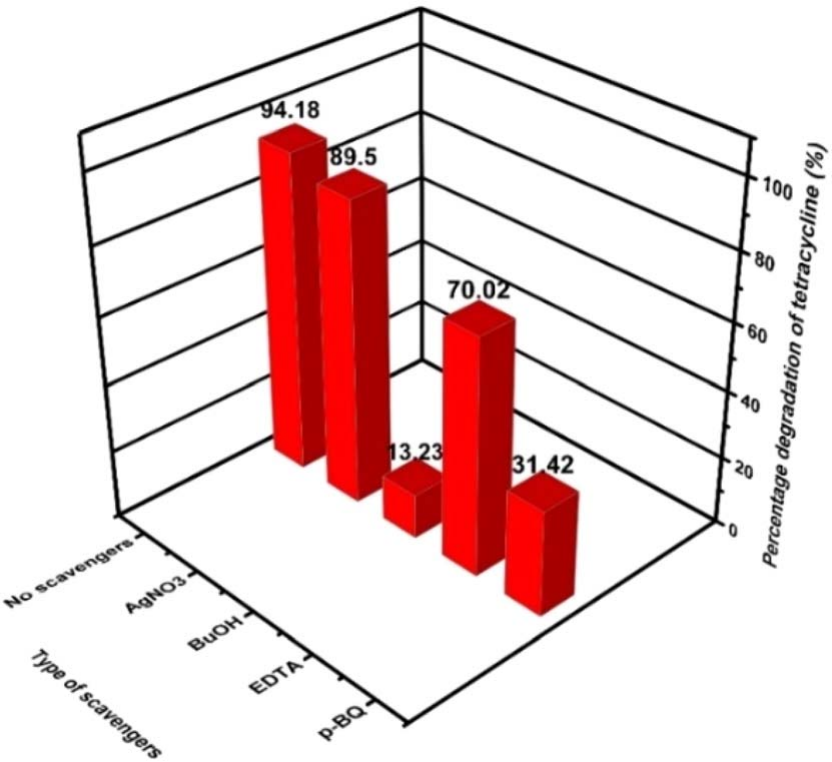
Scavenger effects on TETR degradation.

Radical quenching experiments were conducted to investigate the predominant active species in ZnO/CDs nanocomposites. Compounds Ethylenediamine tetraacetic acid disodium salt (EDTA), silver nitrate (AgNO_3_), *p*-benzoquinone (*p*-BQ), and *tert*-butanol (BuOH) were introduced to TETR solution as a scavenger for protons (h^+^), electrons (e^−^), superoxide radicals (˙O_2_^−^), and for hydroxyl radicals (˙OH) respectively.^48,49^ As displayed in Fig. 6, BuOH exhibited the strongest restraint effect, which was followed by *p*-BQ. The addition of AgNO_3_ give subtle changes in the degradation efficiency of TETR, which means only a small portion of the electrons participated in the photodegradation processes. However, the use of BuOH or *p*-BQ as scavengers for ˙OH or ˙O^2−^, respectively, clearly hampered the rate of degradation, which indicates that ˙OH and (˙O^2−^) are the main active species in the photodegradation system when irradiated with light.

### 3.7. Possible insights into the photocatalytic mechanism of ZnO/CDs composites

**Fig. 7:**
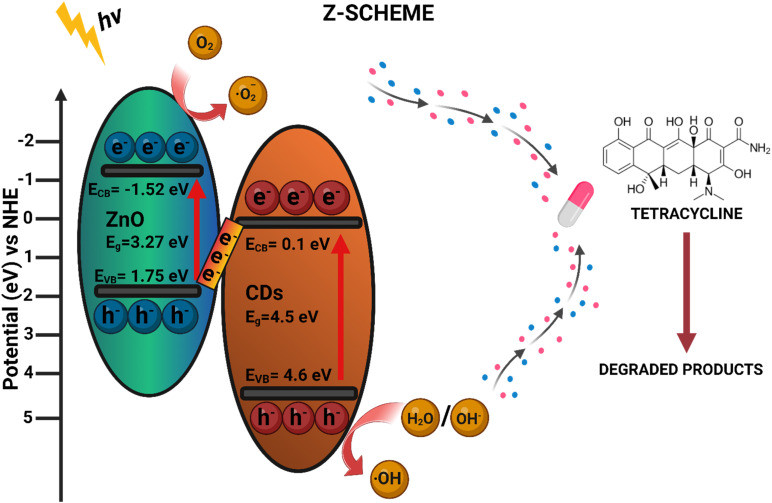
Z-scheme charge transfer mechanism of ZnO/CDs nanocomposite for tetracycline photodegradation.

Upon illumination, both ZnO and CDs absorb photons, leading to the excitation of electrons from the VB to the CB, thereby generating electron–hole pairs in both semiconductors. The Z-scheme (Fig. 7) charge transfer mechanism facilitates the recombination of photogenerated electrons in the CB of CDs with holes in the VB of ZnO. This selective recombination process ensures that the ZnO CB retains highly energetic electrons with strong reducing capability, while the CDs VB preserves holes with potent oxidative power.^50^ Consequently, this spatial charge separation minimizes charge carrier recombination losses and enhances redox reactions, thereby improving the photocatalytic efficiency of the composite.^51^ The preserved electrons in the CB of ZnO possess sufficient reducing power to react with adsorbed oxygen molecules, generating superoxide radicals ˙O^2−^. These radicals further undergo secondary reactions to form hydroxyl radicals (˙OH), which exhibit strong oxidation potential and play a critical role in the degradation of organic pollutants. Meanwhile, the holes retained in the VB of CDs participate in the oxidation of hydroxyl groups or water molecules, generating additional hydroxyl radicals that further contribute to the degradation process. This dual-pathway generation of reactive oxygen species (ROS) enhances the degradation efficiency, leading to the effective breakdown of contaminants into harmless mineralized products. The synergistic charge transfer and ROS generation within the ZnO/CDs nanocomposite contribute to its superior photocatalytic activity compared to pristine ZnO. The efficient utilization of charge carriers not only facilitates rapid redox reactions but also ensures sustained photocatalytic performance. The Z-scheme heterojunction plays a pivotal role in extending charge carrier lifetime, suppressing electron–hole recombination, and promoting the selective participation of charge carriers in oxidation–reduction reactions. The overall mechanism is governed by the following reactions:

3 CD_s_ + hv → e_CB,CDs_^−^ + h_VB,CDs_^+^

4 ZnO + hv → e_CB,ZnO_^−^ + h_VB,ZnO_^+^

5 e_CB,CDs_^−^ + h_VB,CDs_^+^ → recombination

6 O_2_ + H^+^ → ˙OH + OH^−^

7 H_2_O + h_VB,CDs_^+^ → ˙OH + H^+^

8 OH^−^ + h_VB,CDs_^+^ → ˙OH

By leveraging this Z-scheme mechanism, the ZnO/CDs nanocomposites efficiently facilitate charge separation and enhance ROS generation, ultimately leading to improved photocatalytic degradation of organic contaminants.

### 3.8 TETR degradation path ways

**Fig. 8:**
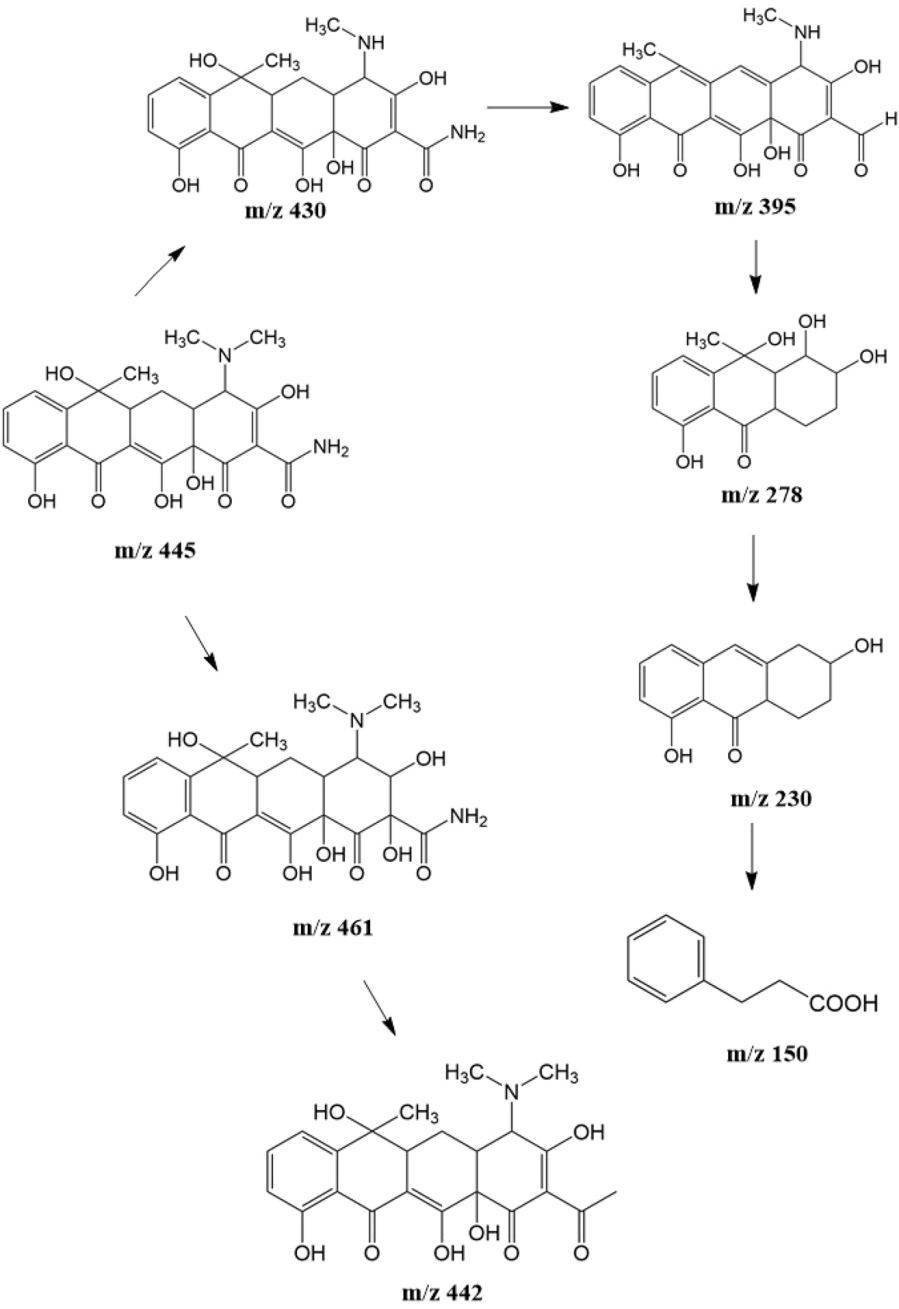
The intermediates product of TETR degradation.

Based on the identified degradation intermediates (Table 2) and predicted reactive sites (ESI, Fig. S5†), a potential photocatalytic degradation pathway for tetracycline (TETR, *m*/*z* 445) over the ZnO/carbon dots photocatalyst is proposed (Fig. 8). The degradation process begins with the transformation of tetracycline into two primary intermediates. The first intermediate, *m*/*z* 430, suggests the loss of a CH_2_ moiety, likely through oxidative decarboxylation or demethylation.^52,53^ The second intermediate, *m*/*z* 461, indicates the addition of an oxygen atom, potentially through hydroxylation or epoxidation, which is commonly observed in oxidative photocatalytic reactions involving reactive oxygen species.^54^ Further degradation leads to the formation of *m*/*z* 442, suggesting a combination of demethylation and oxidation, likely involving the removal of a methyl group (–CH_3_) and the formation of a keto functional group. The subsequent transformations indicate progressive structural degradations. The conversion of *m*/*z* 430 to *m*/*z* 395 suggests the loss of a methyl group (–CH_3_) and an amide functional group (–CONH_2_), likely due to oxidative cleavage of the amide bond, a known degradation pathway under photocatalytic conditions.^55^ The transition from *m*/*z* 395 to *m*/*z* 278 indicates major structural fragmentation, possibly involving ring-opening reactions facilitated by hydroxyl radical attack. The degradation of *m*/*z* 278 to *m*/*z* 230 suggests further cleavage of the tetracycline core, likely through oxidative bond scission.^56^ The final step, *m*/*z* 230 to *m*/*z* 150, represents the formation of a benzoic acid derivative, a common low-molecular-weight breakdown product in the advanced degradation of aromatic compounds.^57^ This transformation may result from decarboxylation and hydroxylation reactions, leading to a stable carboxylated aromatic structure.^51^ These intermediates are expected to undergo further oxidation and mineralization, ultimately leading to the formation of CO_2_ and H_2_O under prolonged photocatalytic conditions. The proposed pathway highlights the efficiency of ZnO/carbon dots nanocomposites in facilitating the stepwise breakdown of tetracycline into progressively smaller and less toxic fragments, contributing to effective environmental remediation.

**Table 2:** Structural information of possible intermediate products identified by LCMS

Formula	*m*/*z*	Proposed structures	Ref.
C_22_H_24_N_2_O_8_	445	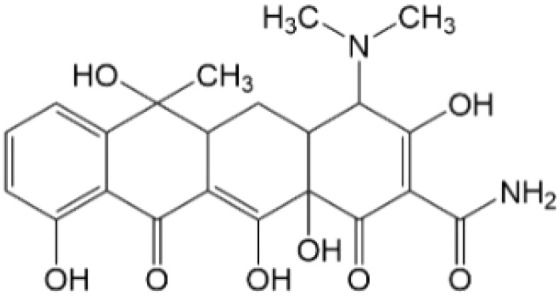	56
C_21_H_17_NO_7_	395	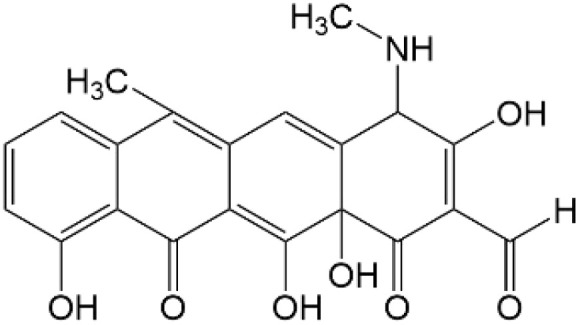	53
C_22_H_26_N_2_O_9_	461	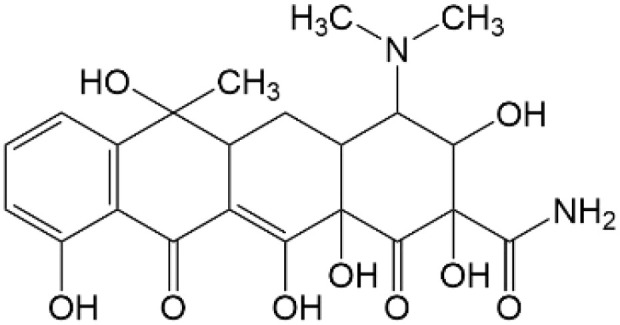	52
C_23_H_25_NO_8_	442	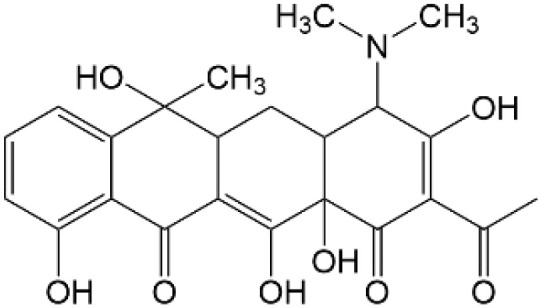	55
C_21_H_22_N_2_O_8_	430	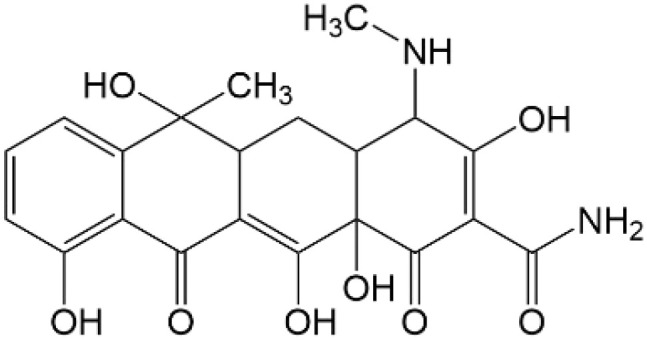	58
C_15_H_18_O_5_	278	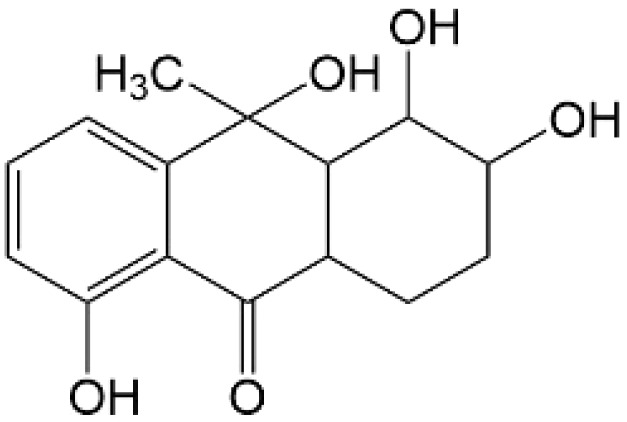	55
C_14_H_14_O_3_	230	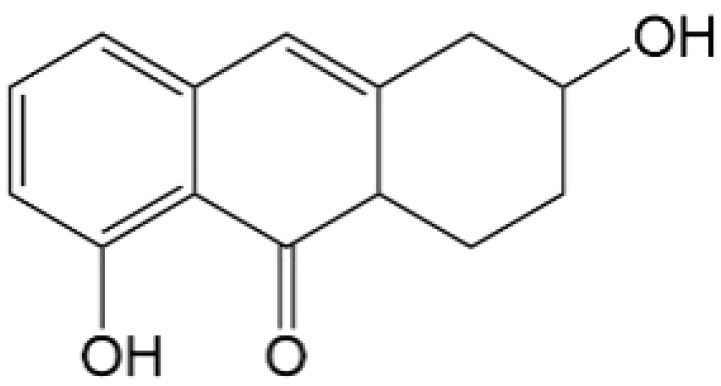	55
C_9_H_10_O_2_	150	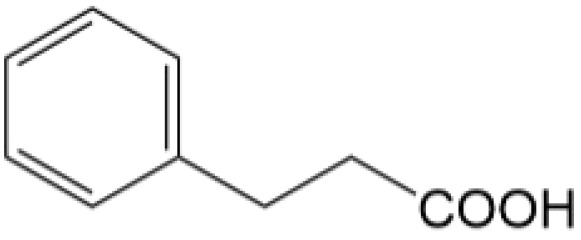	55

### 3.9. Photodegradation of tetracycline

#### 3.9.1. Effect of different sources of light

**Fig. 9:**
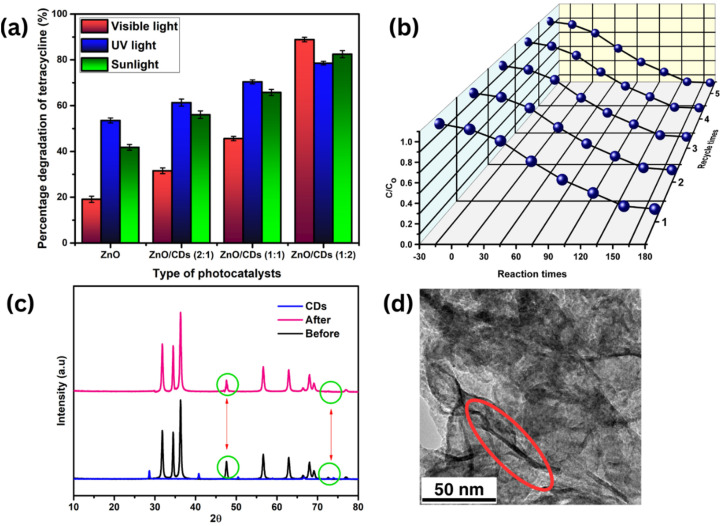
(a) Effect source of light on TETR degradation; (b) reusability test; (c) XRD pattern, and (d) HRTEM image of the recycled ZnO/CDs (1 : 2).

The varying photocatalytic performance of ZnO/CDs nanocomposites under different light sources such as UV, visible, and sunlight can be attributed to the light absorption properties and charge separation efficiency of the materials. Under UV light, ZnO, with its wide bandgap (3.27 eV), efficiently absorbs photons, generating electron–hole pairs that contribute to high degradation efficiency (Fig. 9a). A higher ratio of CDs to ZnO further enhances this process by acting as electron sinks, preventing recombination and improving photocatalytic performance. Under visible light, where ZnO absorption is limited, CDs play a more critical role by absorbing visible light and transferring the resulting photoinduced electrons to ZnO. This electron transfer significantly boosts photocatalytic activity, with the ZnO/CDs 1 : 2 nanocomposite achieving a high degradation efficiency of 86.87%. In contrast, a lower CDs to ZnO content reduces visible light absorption and hinders charge separation, resulting in lower performance of 31.55% as illustrated in Fig. 9a. Under sunlight, which contains both UV and visible light components, the photocatalytic performance is influenced by the combined effects of ZnO's UV absorption and the visible light harvesting ability of carbon dots. For the ZnO/CDs 2 : 1, an efficiency of 58.74% was achieved. These findings align with previous studies by Zhang *et al.* (2015)^59^ and Liu *et al.* (2012),^60^ which highlight the synergistic effects of ZnO and CDs across a broad light spectrum.^54^ The optimized ZnO/CDs ratio in this study similarly enhanced photocatalytic performance under both solar and visible light irradiation.^61^

### 3.10. Recycling and reusability

These findings emphasize the excellent stability and reusability of the ZnO/CDs (1 : 2) photocatalyst, which retained a high degradation efficiency of 85% even after five consecutive cycles (Fig. 9b). Although a slight decrease from the initial 86% efficiency was observed, the photocatalyst demonstrated remarkable durability with minimal structural changes. XRD analysis (Fig. 9c) revealed only minor modifications in the catalyst's structure following repeated recycling, likely attributed to surface alterations or slight degradation due to prolonged exposure to the reaction environment. Additionally, HRTEM analysis confirmed the preservation of the ZnO/CDs heterostructure after multiple cycles, further demonstrating the material's structural integrity (Fig. 9d). Morphological analysis further revealed that the overall structure of the catalyst remained intact following the reusability test, reinforcing its robustness for long-term applications. These results align with previous studies reporting, minimal structural changes in photocatalysts after multiple degradation cycle.^9,10^ Collectively, these observations findings highlight the significant strong potential of ZnO/CDs (1 : 2) nanocomposite as a highly stable and recyclable photocatalyst for sustainable wastewater treatment. To further validate the performance, a comparison with previously reported ZnO and CDs-based photocatalysts for TETR degradation is presented in Table 3. The ZnO/CDs developed in this study achieved a competitive degradation efficiency of 86.6% within 180 minutes, utilizing a low-energy 15 W UV lamp and moderate photocatalyst dosage (1 g L^−1^), outperforming many other systems that required higher catalyst loading or more intense light sources.

**Table 3:** Comparison of tetracycline photodegradation by ZnO-based and CDs-based nanocomposites under different synthesis and operational conditions

Type of photocatalyst	Light source	Dosage of photocatalyst (g L^−1^)	TETR concentration (mg L^−1^)	Degradation efficiency (%)	Ref.
ZnO/CDs	15 W of UV lamp	1	15	86.6% in 180 min	This work
BTiO_2_–ZnO	300 W Xe lamp	0.1	50	63% in 120 min	5
Ag/ZnO@biochar	500 W of Xe lamp	0.01	50	70.3 in 60 min	2
CQDs/TiO_2_	350 W of Xe lamp	0.2	20	62.3% in 120 min	62
CDs/g-C_3_N_4_/MoO_3_	350 W of Xe lamp	0.15	20	88.4% in 90 min	53
CDs/g-C_3_N_4_	350 W of Xe lamp	0.2	20	86% in 120 min	63
TiO_2_/ZnO	UV light	0.5	30	86.3% in 3 h	64

## 4. Conclusions

This study presents a novel Z-scheme ZnO/carbon dots heterojunction, synthesized *via* a simple hydrothermal method, achieving an impressive 86.6% degradation efficiency of tetracycline (TETR). The superior photocatalytic performance is attributed to the efficient charge separation and transfer enabled by the built-in electric field of the Z-scheme heterojunction, where CDs act as both visible-light sensitizers and electron mediators, promoting charge carrier separation and enhancing the generation of reactive oxygen species (˙OH and ˙O_2_^−^) that drive TETR degradation. Energy band structure analysis and photoelectrochemical measurements confirm the successful formation of the Z-scheme heterojunction, providing direct evidence for the proposed charge transfer mechanism. The optimized ZnO/CDs ratio (1 : 2) underscores the significance of interface engineering in maximizing synergistic effects, offering valuable insights for the rational design of advanced photocatalysts. Compared to conventional photocatalysts, the ZnO/CDs composite exhibits superior efficiency and potential long-term stability, highlighting its promise for practical wastewater treatment. This work advances the understanding of charge transfer in Z-scheme photocatalysts and presents a scalable, sustainable approach for antibiotic removal. Future work will focus on evaluating performance in real wastewater systems and exploring its impacts on antimicrobial resistance (AMR), further validating its environmental and practical relevance.

## Author contributions

Wan Nuraishah Wan Ishak: formal analysis, investigation, and writing – original draft; Huey Ling Tan, Norbert Radacsi, Ying Pei Lim: conceptualization, supervision, writing – review and editing, and validation; Noor Fitrah Abu Bakar: formal analysis, supervision, and validation.

## Conflicts of interest

There are no conflicts of interest to declare.

## Supplementary Material

RA-015-D5RA02606G-s001

## Data Availability

The data supporting this article have been included as part of the ESI.†
