# Supplementary material for: Electrochemical characterization of Z-scheme charge transfer in biomass-derived ZnO/carbon dots for efficient tetracycline degradation
Source: RSC Adv. 2025 Jul 14;15(30):24726–38. doi: 10.1039/d5ra02606g (PMC12257474; doi:10.1039/d5ra02606g)
Supplement: RA-015-D5RA02606G-s001 [file RA-015-D5RA02606G-s001.pdf]

Supplementary Information (SI) for RSC Advances.
This journal is © The Royal Society of Chemistry 2025

Supplementary Information

Electrochemical Characterization of Z-Scheme Charge Transfer in Biomass-Derived ZnO/Carbon Dots for Efficient Tetracycline Degradation

Wan Nuraishah Wan Ishak[a], Huey Ling Tan[a]*, Noor Fitrah Abu Bakar[a], Norbert Radacsi [b,c,d]*, and Ying Pei Lim[a]

[a]Faculty of Chemical Engineering, Universiti Teknologi MARA, 40450 Shah Alam, Selangor, Malaysia, [b]School of Engineering, Institute for Materials and Process, The University of Edinburgh, King's Buildings, Edinburgh EH9 3FB, UK School of Engineering, [c]Institute for Bioengineering, The University of Edinburgh, Mayfield Road, Edinburgh, EH9 3JL, United Kingdom, [d] Centre for Cardiovascular Science, The Queen's Medical Research Institute (QMRI), University of Edinburgh, BioQuarter, 47 Little France Crescent, Edinburgh, EH16 4TJ, United Kingdom

Email addresses:

(Wan nuraishah Wan Ishak) 2022288228@student.uitm.edu.my

* (Huey Ling Tan) hueyling@uitm.edu.my

(Noor Fitrah Abu Bakar) fitrah@uitm.edu.my

* (Norbert Radacsi) N.Radacsi@ed.ac.uk

(Ying Pei Lim) yingpei@uitm.edu.my

*Corresponding author. Tel.: +60 (0) 3 5543 6310. E-mail address: hueyling@uitm.edu.my

*Joint corresponding author. Tel.: +44 (0) 131 651 7112. E-mail address: n.radacsi@ed.ac.uk

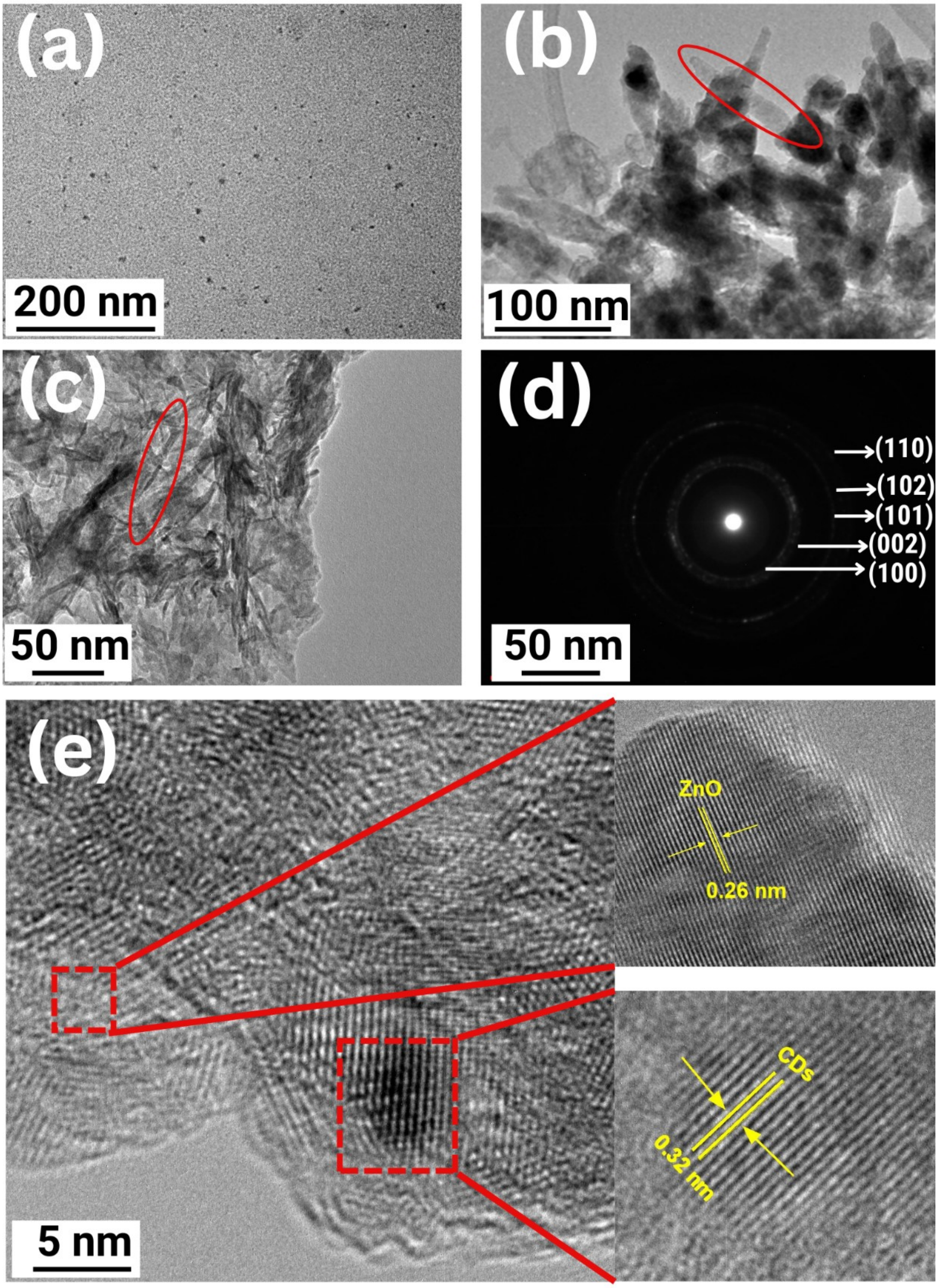

Fig. S1 HRTEM images (a) CDs, (b) ZnO, (c) ZnO/CDs (1:2); SAED patterns of ZnO/CDs: Lattice fringes from HRTEM analysis of ZnO/CDs nanocomposite

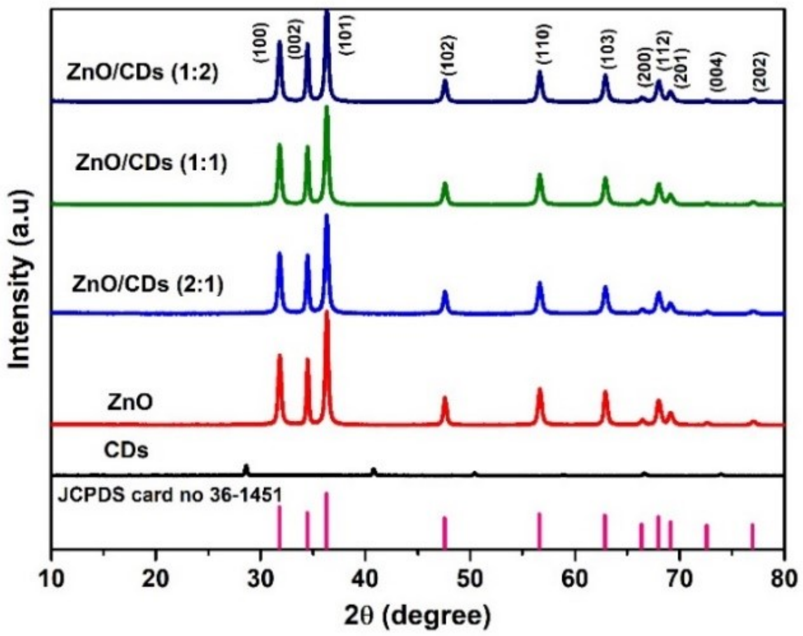

Fig. S2 XRD patterns of ZnO, carbon dots (CDs), and ZnO/CDs composites (2:1, 1:1, 1:2)

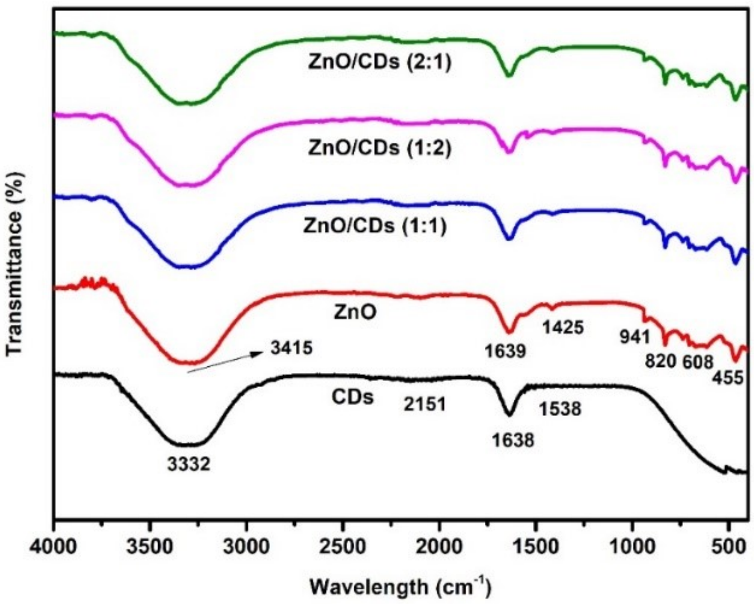

Fig. S3 FTIR spectrum of CDs, ZnO nanoparticles and ZnO/CDs composites.

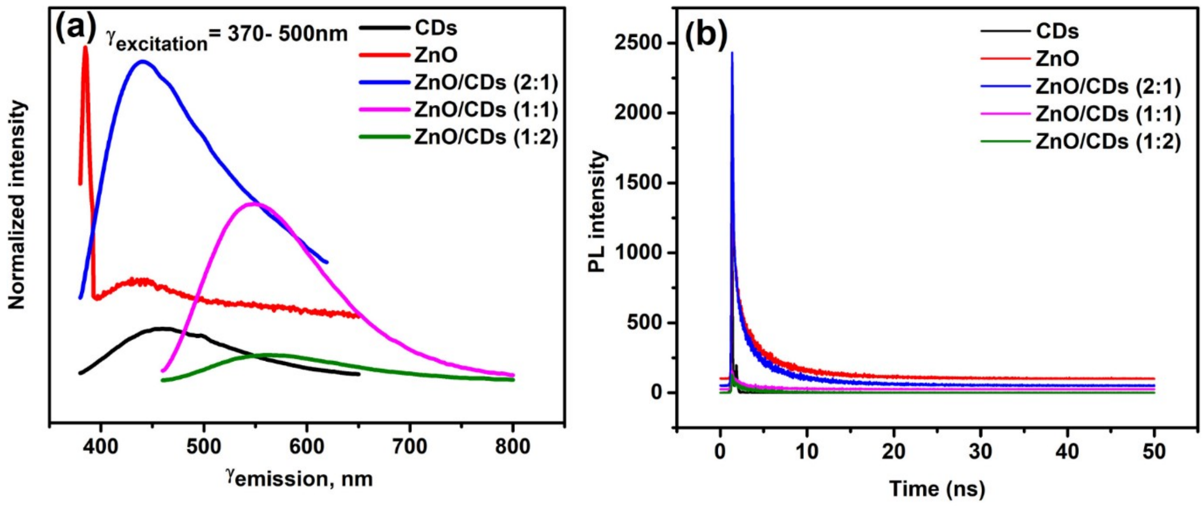

Fig. S4 (a) The PL spectra of CDs, ZnO nanoparticles and, ZnO/CDs nanocomposites at different excitation wavelength and (b) TRPL of CDs, ZnO nanoparticles and ZnO/CDs composites

Table S1. TRPL Decay rate of ZnO nanoparticles and ZnO/CDs composites

Sample	τ_1 (ns)	B_1 (%)	τ_2 (ns)	B_2 (%)	τ_3 (ns)	B_3 (%)	τ_{avg} (ns)
**CDs**	0.0456	0.096	1.142	0.002	4.416	0.001	2.013
**ZnO**	0.149	0.009	1.657	0.002	6.370	0.001	4.198
**ZnO/CDs (2:1)**	0.0931	0.020	1.367	0.003	5.999	0.001	3.492
**ZnO/CDs (1:1)**	0.146	0.072	2.467	0.007	9.209	0.001	3.485
**ZnO/CDs (1:2)**	0.177	0.017	3.317	0.491	3.3176	0.489	2.084

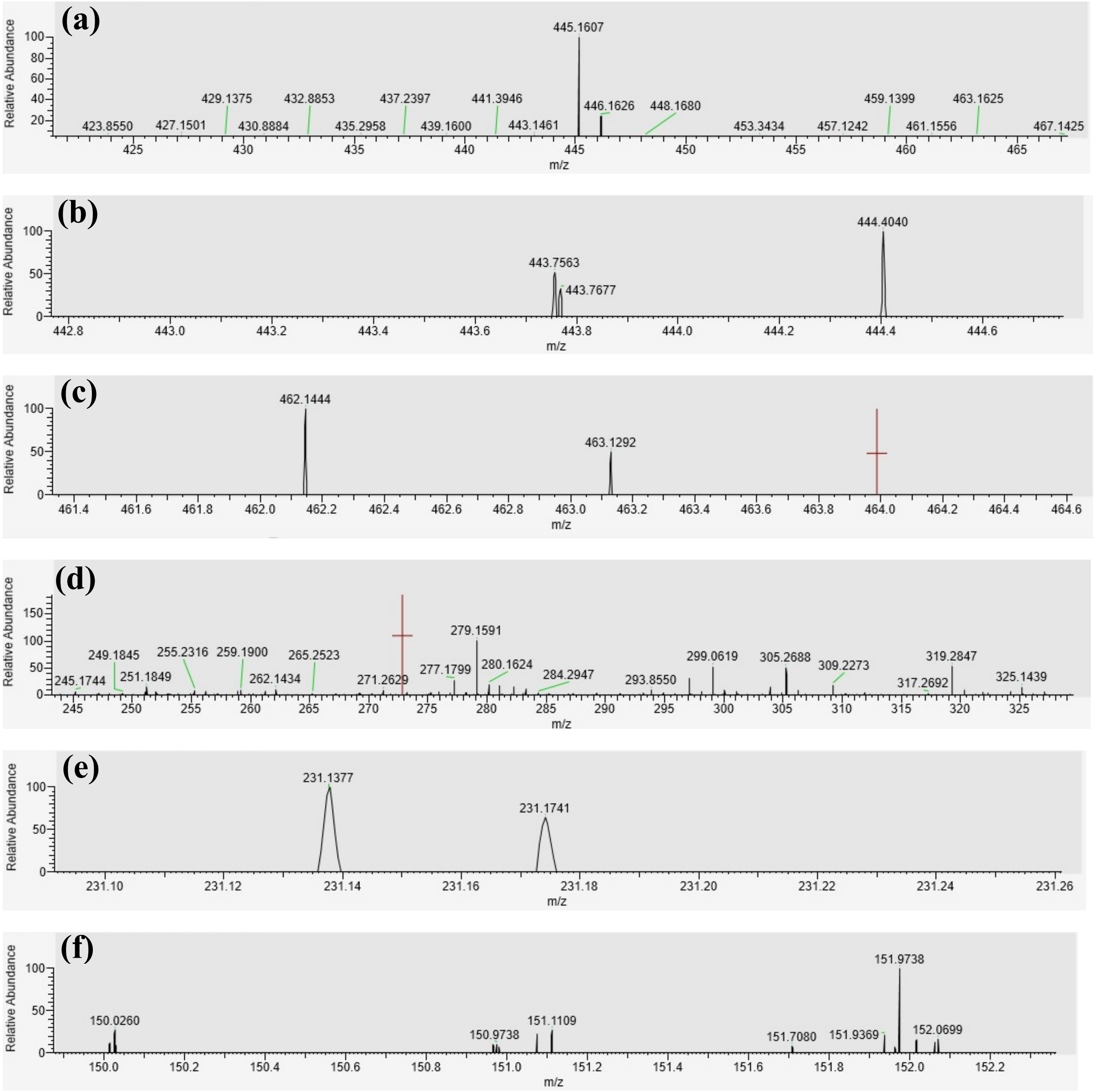

Fig S5 Liquid chromatography mass spectrometry analysis degradation of proposed structure intermediates product for (a) m/z 445, (b) m/z 442, (c) m/z 461, (d) m/z 278, (e) m/z 230 and, (f) m/z 150.
